# Pathogen Profiles in Outpatients with Non-COVID-19 during the 7th Prevalent Period of COVID-19 in Gunma, Japan

**DOI:** 10.3390/microorganisms11092142

**Published:** 2023-08-24

**Authors:** Hirokazu Kimura, Yuriko Hayashi, Masanari Kitagawa, Miwa Yoshizaki, Kensuke Saito, Kazuhiko Harada, Kaori Okayama, Yusuke Miura, Ryusuke Kimura, Tatsuya Shirai, Kiyotaka Fujita, Suguru Machida, Kazuto Ito, Isao Kurosawa

**Affiliations:** 1 Department of Health Science, Gunma Paz University Graduate School of Health Sciences, Takasaki-shi 370-0006, Gunma, Japan; hayashi@paz.ac.jp (Y.H.); k_harada@bishinkai.or.jp (K.H.); okayama@paz.ac.jp (K.O.); miura@paz.ac.jp (Y.M.); fujita@paz.ac.jp (K.F.); 2 Advanced Medical Science Research Center, Gunma Paz University Research Institute, Shibukawa-shi 377-0008, Gunma, Japan; m2220015@gunma-u.ac.jp (R.K.); dw57td@bma.biglobe.ne.jp (T.S.); kzito@gunma-u.ac.jp (K.I.); 3 Project Department, Takara Bio Inc., Kusatsu-shi 525-0058, Shiga, Japan; kitagawam@takara-bio.co.jp (M.K.); yoshizakim@takara-bio.co.jp (M.Y.); saitokx@takara-bio.co.jp (K.S.); 4 Kurosawa Hospital, Takasaki-shi 370-1203, Gunma, Japan; s_machida@bishinkai.or.jp (S.M.); i_kurosawa@bishinkai.or.jp (I.K.); 5 Department of Bacteriology, Graduate School of Medicine, Gunma University, Maebashi-shi 371-8514, Gunma, Japan

**Keywords:** pathogen profiles, non-COVID-19 outpatients, next-generation sequencer, bioinformatics

## Abstract

The identification of pathogens associated with respiratory symptoms other than the novel coronavirus disease 2019 (COVID-19) can be challenging. However, the diagnosis of pathogens is crucial for assessing the clinical outcome of patients. We comprehensively profiled pathogens causing non-COVID-19 respiratory symptoms during the 7th prevalent period in Gunma, Japan, using deep sequencing combined with a next-generation sequencer (NGS) and advanced bioinformatics technologies. The study included nasopharyngeal swabs from 40 patients who tested negative for severe acute respiratory syndrome coronavirus type 2 (SARS-CoV-2) using immuno-chromatography and/or quantitative reverse transcription polymerase chain reaction (qRT-PCR) methods. Comprehensive pathogen sequencing was conducted through deep sequencing using NGS. Additionally, short reads obtained from NGS were analyzed for comprehensive pathogen estimation using MePIC (Metagenomic Pathogen Identification Pipeline for Clinical Specimens) and/or VirusTap. The results revealed the presence of various pathogens, including respiratory viruses and bacteria, in the present subjects. Notably, human adenovirus (HAdV) was the most frequently detected virus in 16 of the 40 cases (40.0%), followed by coryneforms, which were the most frequently detected bacteria in 21 of the 40 cases (52.5%). Seasonal human coronaviruses (NL63 type, 229E type, HKU1 type, and OC43 type), human bocaviruses, and human herpesviruses (human herpesvirus types 1–7) were not detected. Moreover, multiple pathogens were detected in 50% of the subjects. These results suggest that various respiratory pathogens may be associated with non-COVID-19 patients during the 7th prevalent period in Gunma Prefecture, Japan. Consequently, for an accurate diagnosis of pathogens causing respiratory infections, detailed pathogen analyses may be necessary. Furthermore, it is possible that various pathogens, excluding SARS-CoV-2, may be linked to fever and/or respiratory infections even during the COVID-19 pandemic.

## 1. Introduction

In Japan, a novel coronavirus disease (COVID-19), caused by severe acute respiratory syndrome coronavirus type 2 (SARS-CoV-2), emerged in January 2020 [1]. Subsequently, the disease experienced eight prevalence surges over the course of three years in Japan. Particularly noteworthy is the seventh prevalence period (surge) of COVID-19, which occurred from July to November 2022, during which approximately 13 million people were reported as COVID-19 cases in Japan. At the peak of the 7th prevalence period, SARS-CoV-2 was detected in around 50% of cases presenting with fever and/or respiratory symptoms [2]. Gunma prefecture is located in the middle of Japan, about 100 km away from Tokyo [3]. Due to such locations, similar patterns of COVID-19 prevalence were observed between Gunma and Tokyo, although the disease incidence differed [4].

In general, various respiratory viruses may exhibit seasonal prevalence [5]. For example, seasonal influenza and respiratory syncytial virus (RSV) infections tend to peak from autumn to winter in the northern hemisphere, although the exact reason for this is not known [6]. Moreover, when emerging virus infections occur, the prevalence patterns of other seasonal infections may fluctuate [5,7]. In fact, the emergence of COVID-19 significantly impacted the prevalence of seasonal influenza and other respiratory infections [7].

Due to the COVID-19 vaccine campaign and pathogenicity changes in the causative agent, such as the omicron subvariants of the severe acute respiratory syndrome coronavirus type 2 (SARS-CoV-2), the clinical manifestations of COVID-19 have undergone significant changes compared to previous strains such as the delta variant [8]. Indeed, the incidence of pneumonia and other severe respiratory symptoms [i.e., adult respiratory distress syndrome (ARDS)] due to the disease drastically decreased in Japan and other developed countries [9]. Indeed, the incidence of pneumonia and other severe respiratory symptoms, such as ARDS, has drastically decreased in Japan and other developed countries [10]. Most cases of COVID-19 now present with symptoms such as mild to moderate fever, sore throat, rhinitis, and cough without lower respiratory symptoms. These symptoms may resemble those of the common cold [11]. Furthermore, respiratory specimens, such as nasopharyngeal swabs, may contain a mixture of pathogenic and non-pathogenic microorganisms [12]. In certain instances, colonized respiratory pathogens may be responsible for asymptomatic infections [13]. For instance, *Streptococcus pyogenes* can establish colonization, yet it can also lead to pyogenic tonsillitis, and in severe cases, it can result in systemic invasive infections [14]. Thus, it may be challenging to differentiate various respiratory pathogens based solely on the respiratory symptoms observed in COVID-19 cases [15]. However, accurate pathogen diagnosis is important for estimating clinical outcomes in patients. 

Deep sequencing methods combined with a next-generation sequencer (NGS) have been developed to comprehensively detect pathogens [16]. In addition, comprehensive pathogen estimation tools that utilize short reads from NGS, such as MePIC (Metagenomic Pathogen Identification Pipeline for Clinical Specimens) and VirusTap, have also been developed [17]. In this study, we employed these methods to estimate the presence of different pathogens among outpatients exhibiting various symptoms, such as fever and/or respiratory symptoms (cough, phlegm, and sore throat), unrelated to COVID-19 during the 7th prevalence period (September to October 2022) in Gunma, Japan.

## 2. Materials and Methods

### 2.1. Subjects and Ethics Status

From 20 September to 28 October 2022, we collected 40 nasopharyngeal swabs (NPS) from outpatients with non-COVID-19 who provided written informed consent. These patients had been diagnosed as negative for SARS-CoV-2 using a quantitative reverse transcription polymerase chain reaction (qRT-PCR) method. The samples were stored at −80 °C until use. The demographic data are shown in Table 1, and detailed data on the subjects are provided in Supplementary Table S1. Furthermore, during the investigation period, the detection rate of SARS-CoV-2 in the hospital among outpatients was approximately 40% as well as other hospitals locating in Tokyo [2]. The study protocol was approved by the Ethics Committee on Human Research of Paz University (approval no. Paz22-29) on 15 November 2022, and the protocols were carried out in accordance with the approved guidelines.

### 2.2. Deep Sequencing and Pathogen Estimation

DNA and RNA were extracted from the NPS samples using an available kit (QIAamp Viral RNA Kits, Venlo, Netherlands). The preparation of libraries for deep sequencing was previously described [17,18,19]. Deep sequencing was performed using a next-generation sequencer (iSeq100, Illumina, San Diego, CA, USA). Short reads (approximately 150–250 nucleotides) from deep sequencing were analyzed by MePIC and/or VirusTap [17]. Pathogen estimation was conducted as previously described [17,18,19]. Additionally, to determine the genotypes and/or subgroups, we generated partial contig sequences from the short reads and identified virus genotypes, including enterovirus, RSV, and human rhinovirus (HRV), using BLAST (https://blast.ncbi.nlm.nih.gov/Blast.cgi, accessed on 4 June 2023) [20]. Furthermore, the pathogenicity of the *corynebacterium* was determined and confirmed based on the report of Oliveira et al. [21].

## 3. Results

### 3.1. Demographic Data

As shown in Table 1 and Table S1, the mean age of the present subjects was 41.7 years (ranging from 2 (minimum) to 79 (maximum) years, and the rate of males and females was similar (23 cases vs. 17 cases). Moreover, various symptoms regarding respiratory infections such as fever (mild to high fever), sore throat, cough, phlegm, and headache. In the present subjects, fever was the most frequent symptom in 31 of 40 cases (77.5%), followed by sore throat (55.0%), malaise (45.0%), cough (32.5%), and headache (20.0%). No pneumonia case was seen in the present cases.

**Table 1:** Demographic data in this study.

	All Subjects	Male Subjects	Female Subjects
Case numbers (%)	40	23 (57.5)	17 (42.5)
Age (mean ± SD)	41.7 ± 19.5	38.4 ± 19.5	46.1 ± 19.2
Range of the age (years)	2–79	2–72	20–79

### 3.2. Individually Demographic Data and Pathogen Profiles

We also individually show the demographic data and pathogen profiles of the present subjects in Table 2. Overall, the subjects manifested various respiratory symptoms, including sore throat, cough, and phlegm. Most of the subjects also revealed other symptoms such as fever, headache, and malaise. Among them, various pathogens, including viruses and bacteria, were detected in 92.5% (37/40 cases) of the patients.

Next, we present the demonstration of Table 2 and Table 3. As depicted in Table 2, various pathogens, including respiratory viruses and bacteria, were detected in the present subjects. Among them, HAdV-C was the most frequently detected virus, found in 8 out of 40 cases (20.0%), followed by RSV (6/40 cases, 15.0%) and HRV (4/40 cases, 10.0%). No other respiratory and/or fever-associated viruses, including seasonal human coronaviruses (NL63 type, 229E type, HKU1 type, and OC43 type), human bocaviruses, and human herpesviruses (human herpesvirus types 1–7), were detected. In addition, numerous coryneform species were detected in 21 out of 40 cases (52.5%), with *Moraxella catarrhalis* (5/40 cases, 12.5%) being the second most common. Among the cases, 9 (9/40 cases, 22.5%) had only a virus detected, and the predominant virus was HAdV-C (6/9 cases, 66.7%). Similarly, among the cases, 15 (15/40 cases, 37.5%) had only bacteria detected, and the dominant bacteria were coryneforms (9 cases, 9/15 cases, 60.0%).

As shown in Table 3, we summarized the pathogen profiles of coryneforms in the present cases. Pathogenic coryneforms such as *C. accolens*, *C. pseudotuberculosis*, and *C. striatum* were the most frequently detected in 14 out of 40 cases (35.0%), followed by untypable species (11/40 cases, 27.5%). Furthermore, as indicated in Table 2 and Table 3, coryneforms (20/23 cases, 87.0%) were the most commonly detected pathogens in cases with multiple pathogens, followed by HAdV-C (11/23 cases, 47.8%). In these cases, multiple pathogens were detected, although the number of NGS short reads for HAdV-C was less than 10 counts. Moreover, in 12 cases, both viruses and bacteria were detected. The cases with the highest number of bacteria detected exhibited a high number of short reads. These results suggest that various respiratory viruses and bacteria were associated with non-COVID-19 cases, even during the COVID-19 pandemic period.

### 3.3. Non-Pathogenic Coryneforms Detected in the Present Cases 

We provide an overview of the non-pathogenic coryneforms identified in the current study (Table 4). In addition, detailed profiles of non-pathogenic coryneforms for individual cases are shown in Table S1. Among the cases examined, the most frequently detected coryneforms were *C. segmentosum*, *C. kefirresidentii*, and *C. tuberculostearicum*, with these species being identified in over 70% of cases. Other non-pathogenic coryneforms, including *C. sanguinis* and *C. simulans*, were also present in more than 50% of cases. Furthermore, a variety of other non-pathogenic coryneforms were also identified (Table 4 and Table S1). These findings suggest that diverse non-pathogenic coryneforms colonized and altered the composition of the respiratory bacterial flora in the cases under study.

## 4. Discussion

To comprehensively detect and estimate various pathogens in the outpatients with non-COVID-19 cases, we performed deep sequencing. Moreover, we estimated pathogens using short reads from deep sequencing and advanced bioinformatics technologies. As a result, various viruses and bacteria may be associated with the apparent infections in the present cases. Furthermore, multiple pathogens may be associated with these cases. The results suggest that various pathogens may be associated even during the COVID-19 pandemic period (7th prevalent period) in Gunma Prefecture, Japan. Therefore, for accurate pathogenic diagnosis in patients with respiratory infections, detailed pathogen analyses may be needed [22].

The deep sequencing method combined with NGS enables us to comprehensively analyze the pathogen genomes in various clinical specimens, including NPS. In general, the deep sequencing method provides numerous short reads. These short reads certainly contain both pathogen and human sequences. To estimate pathogens in the samples, two further analyses may be needed, as follows: Removing human genome sequences and comprehensive pathogen genome analyses using suitable bioinformatics tools. MePIC and VirusTap promptly provide these requirements [14,15,16]. Therefore, we used these advanced bioinformatics tools in this study. As a result, we could comprehensively estimate various pathogens in non-COVID-19 patients. This may be the first observation.

In this study, various respiratory pathogens were detected (Table 1, Table 2 and Table 3). It is suggested that these pathogens are commonly detected in various respiratory diseases, including the common cold, tonsillitis, pharyngitis, laryngitis, croup, bronchitis, bronchiolitis, and pneumonia [23,24,25,26]. Furthermore, these illnesses may be complicated by various symptoms other than respiratory symptoms, such as fever, malaise, and headache [27]. The present cases also manifested not only these typically aforementioned respiratory symptoms but also non-respiratory symptoms (Table 2). Thus, our clinical data may be compatible with other findings.

In this study, the most detected virus was HAdV-C. Currently, there are over 70 HAdV genotypes, differentiated by phylogeny into seven species (HAdV-A–G) [28,29]. Of them, HAdV-C includes HAdV 1, 2, 5, 6, and 57 types, and these viruses were frequently detected in patients with respiratory infections, such as the common cold, tonsillitis, pharyngoconjunctival fever, pharyngitis, and laryngitis [30]. Next, RSV is not only a major causative agent for respiratory infections but also causes reinfection in all-aged people [31,32]. RSV is classified into two subgroups (RSV-A and B), and RSV-A is a more dominant type than RSV-B [33,34,35]. In Japan, RSV-A was mainly detected during the investigation period of the present study (September to October 2022) [36]. Furthermore, HRV is classified into 3 species (HRV-A, B, and C), and these include numerous genotypes [37]. Of them, HRV-A21, A24, and A61 are reportedly dominant genotypes. These viruses may also cause various respiratory illnesses, as well as HAdV and RSV. Furthermore, it is suggested that EV-D68 is also associated with various respiratory infections caused by the aforementioned viruses [38]. However, the troublesome aspect of these viruses is the occurrence of asymptomatic infections [39,40]. All the present subjects showed infectious disease-related clinical sign(s) (Table 2), and around 20% (9/40 cases) of the present cases were detected with the virus alone. Therefore, these detected viruses may be responsible for the illness in these cases, although there were two cases where multiple viruses were detected. In these cases, it may be unclear which virus caused the disease or both.

Other respiratory viruses, such as human bocaviruses and seasonal human coronaviruses (NL63 type, 229E type, HKU1 type, and OC43 type), have been consistently detected in Japan [41]. However, these pathogens were not identified in the current cases (Table 1 and Table 2). These respiratory pathogens typically exhibit prevalence and detectability during the winter season (December to February) in our country [41]. Moreover, they tend to be predominantly detected among infants (less than 5 years old) [42]. Furthermore, the investigation period for this study encompassed the first half of autumn (September to October 2022), with only two infants included among the present cases. Such factors may be linked to the absence of detection of these viruses in the present study. Various human herpesviruses (human herpesvirus types 1–7) are known to be associated not only with rash and fever syndromes but also with respiratory illnesses [43]. These viruses are capable of causing persistent infections and do not consistently display seasonal prevalence patterns [44]. Nevertheless, the reasons for the non-detection of these viruses in the present cases remain unclear.

Next, over 35% (15/40 cases) of the cases were detected with bacteria alone (Table 2). Among them, mono-species pathogenic bacteria were detected in six cases, while multiple-species pathogenic bacteria were detected in nine cases. Moreover, pathogenic viruses and bacteria were detected in 13 cases (32.5%, 13/40) (Table 2). Thus, these bacteria may be the pathogens responsible for these cases. In these cases, it may also be unclear which pathogen caused the disease at all.

Until now, over 100 species of *Corynebacterium* have been identified, and most are part of the normal flora of human skin and mucous membranes [45]. Among them, many pathogenic coryneforms (non-diphtheria *Corynebacterium*) have been confirmed [46]. For example, *C. ulcerans* and *C. pseudodiphtheriticum* can produce potent exotoxins, i.e., diphtheria toxin and/or phospholipase D. These exotoxins can be associated with various respiratory infections [21]. Moreover, other coryneforms (non-producing diphtheria toxin and/or phospholipase D), including *C. accolens*, *C. striatum*, *C. minutissimum*, *C. propinquum*, *C. aurimucosum*, *C. camporealensis*, *C. pseudodiphtheriticum*, *C. jeikeium*, and *C. urealyticum,* were found to be pathogenic, and these may also be associated with respiratory illnesses [21]. In the present cases, these pathogenic coryneforms were most frequently detected (Table 3). 

Over a considerable period, despite the identification of numerous coryneforms, their pathogenicity remained unknown [21]. Recent studies, integrating clinical, genetic, and proteomics data, are now elucidating the pathogenic potential of various coryneforms [21]. Among these, next-generation sequencing (NGS) data coupled with bioinformatics not only furnish precise genome information but also reveal the phylogeny of the coryneforms [47]. Furthermore, proteomics data offer insights into the profiles of coryneform-producing proteins, including exotoxins [48]. Such comprehensive data hold promise for distinguishing between pathogenic and non-pathogenic coryneforms [21]. In the present study, we assessed the pathogenicity of coryneforms using state-of-the-art technology. Consequently, we could gauge the pathogenicity of coryneforms identified in the current cases by harnessing NGS in tandem with advanced bioinformatics technologies (MePIC plus VirusTap). This dataset might represent a pioneering endeavor to unravel the profiles of non-pathogenic and pathogenic coryneforms within the nasopharyngeal bacterial flora. Notably, certain pathogenic coryneforms might also be detectable in healthy subjects [21], while the cases under scrutiny exhibited diverse respiratory symptoms (Table 1). In certain instances within this study, the identified coryneform might not be definitively classified as a pathogen (for example, case 36 in Table 1) [21]. However, the epidemiological links between these bacteria and respiratory ailments could remain incompletely understood. Thus, these findings could be groundbreaking, potentially contributing to an enhanced comprehension of these associations. It is acknowledged, however, that the scale of the present study might be limited (40 cases). Therefore, further and larger studies using deep sequencing and advanced bioinformatics to exactly understand the etiology of the various coryneforms may be needed. In general, various coryneforms are sensitive to most antibiotics, such as macrolide antibacterial drugs [49]. Based on these studies, including the present study, strategies for the use of antibiotics against respiratory infections may need to be altered similarly to what is shown in the present cases.

In this study, the investigation period was autumn in Japan (September to October 2022). It is also suggested that the seasonally prevalent patterns of some pathogens, such as RSV and influenza, changed due to the emergence of COVID-19 [7]. In Japan, RSV sharply decreased in 2020, when the COVID-19 pandemic began [7]. This phenomenon is assumed to be caused by measures to prevent the spread of the COVID-19 infection, such as restricting activities and wearing masks. However, in 2021, there was an increase in RSV-positive cases, and in 2022, an epidemic period was detected at the same time as in 2019 [50]. Indeed, the results suggest that the seasonal pattern of the epidemic may not be changing. Taken together, using deep sequencing combined with NGS technology to predict the pathogens of respiratory infections can be very useful, not only for accurate pathogen confirmation/diagnosis but also for treatment strategies with antibiotics.

## 5. Conclusions

We comprehensively detected various pathogens in non-COVID-19 cases using deep sequencing combined with advanced bioinformatics technologies. As a result, various pathogens may be associated with the apparent infections in the present cases. Moreover, multiple pathogens may be associated with these cases. The results suggest that various pathogens may be associated even during the COVID-19 pandemic period (7th prevalent period) in Gunma Prefecture, Japan. Furthermore, these findings may change strategies for the treatment of respiratory illnesses. Therefore, for an accurate pathogenic diagnosis in patients with respiratory infections, detailed pathogen analyses may be needed.

## Figures and Tables

**Table 2:** Individually demographic data and pathogen profiles in the present subjects.

Case No.	Age	Gender	Onset Date	Sampling Date	Symptoms	Estimated Pathogens
1	29	F	18 September 2022	20 September 2022	Fever, sore throat, cough, headache	HRV-A24 (49) HAdV-C (2)
2	20	F	19 September 2022	20 September 2022	Fever, malaise, headache	HAdV-C (1) *C. accolens* (16) *C. pseudotuberculosis* (6) *C. minutissimum* (4)
3	75	F	17 September 2022	20 September 2022	Fever, malaise, cough, swollen throat	HAdV-C (4) *C. propinquum* (1176) Untypable *corynebacterium* (200) *C. accolens* (120) *C. pseudotuberculosis* (69) *C. striatum* (52)
4	79	F	19 September 2022	20 September 2022	Fever, cough, phlegm, sore throat, stuffy	Not estimable
5	25	F	19 September 2022	21 September 2022	Sore throat, headache	*C. accolens* (6) *C. minutissimum* (4) *C. pseudotuberculosis* (4)
6	31	M	22 September 2022	24 September 2022	Fever, malaise, sore throat, headache	*C. propinquum* (84) *C. accolens* (39) Untypable *corynebacterium* (31) *C. pseudotuberculosis* (7) *C. striatum* (4) *C. jeikeium* (2) *C. minutissimum* (2)
7	56	F	22 September 2022	24 September 2022	Fever, malaise, lymphadenopathy under the ear	*Streptococcus pyogenes* (2)
8	69	M	20 September 2022	24 September 2022	Fever, malaise, headache, cough, phlegm, sneeze	HRV-A61 (8) HAdV-C (2) RSV-A (2) *Moraxella catarrhalis* (2222)
9	72	M	23 September 2022	24 September 2022	Fever, malaise, headache, cough	RSV-A (225)
10	39	M	23 September 2022	24 September 2022	Fever, nausea, chest pain, back pain	Not estimable
11	47	F	26 September 2022	27 September 2022	Fever, malaise, headache, stuffy nose	HAdV-C (2) *C. propinquum* (2) *C. accolens* (1)
12	52	M	28 September 2022	28 September 2022	Fever, cough, diarrhea	RSV-A (2) *C. accolens* (1)
13	46	F	26 September 2022	28 September 2022	Fever, diarrhea	HAdV-C (2) *C. camporealensis* (2) *Staphylococcus aureus* (74)
14	27	F	24 September 2022	28 September 2022	Fever	RSV-A (10) *C. accolens* (44) *C. minutissimum* (13) *C. pseudotuberculosis* (11) Untypable *corynebacterium* (4) *C. striatum* (2) *Staphylococcus aureus* (44)
15	33	M	27 September 2022	30 September 2022	Sore throat	HAdV-C (4)
16	33	F	30 September 2022	30 September 2022	Fever, malaise	HAdV-C (2) RSV-A (2) *C. accolens* (1) *C. striatum* (1)
17	46	M	29 September 2022	30 September 2022	Sore throat	*C. propinquum* (80) Untypable *Corynebacterium* (27) *C. pseudotuberculosis* (4) *C. striatum* (3) *C. minutissimum* (2)
18	15	M	2 October 2022	3 October 2022	Fever, sore throat	HRV-A24 (4642) *C. pseudotuberculosis* (22) *C. accolens* (17) *C. minutissimum* (11) *C. pseudodiphtheriticum* (8) *C. aurimucosum* (6) *C. striatum* (6) Untypable *corynebacterium* (7) *C. urealyticum* (1) *C. jeikeium* (1)
19	63	M	2 October 2022	3 October 2022	Sore throat	Not estimable
20	53	F	3 October 2022	3 October 2022	Fever	*C. accolens* (35) *C. pseudotuberculosis* (9) *C. pseudodiphtheriticum* (6) Untypable *corynebacterium* (6) *C. minutissimum* (5) *C. striatum* (5) *C. aurimucosum* (1)
21	22	F	4 October 2022	5 October 2022	Fever, sore throat, cough	HRV-A21 (747)
22	2	M	6 October 2022	7 October 2022	fever, cough	*Moraxella catarrhalis* (14788)
23	63	M	10 October 2022	11 October 2022	sore throat	*Staphylococcus aureus* (177)
24	31	M	10 October 2022	11 October 2022	Fever, malaise, sore throat, headache	*C. pseudodiphtheriticum* (50) *C. propinquum* (25) Untypable *corynebacterium* (17) *C. accolens* (15) *C. pseudotuberculosis* (11) *C. aurimucosum* (4) *C. camporealensis* (2) *C. striatum* (1)
25	54	M	7 October 2022	11 October 2022	Fever, malaise, sore throat	HAdV-C (2)
26	34	F	12 October 2022	12 October 2022	Fever, cough, sore throat	HAdV-C (2) *C. striatum* (17) *C. pseudotuberculosis* (11) *C. minutissimum* (8) Untypable *corynebacterium* (1) *C. camporealensis* (1)
27	20	M	13 October 2022	14 October 2022	Fever, malaise, sore throat, swollen throat	HAdV-C (2) *C. minutissimum* (5) *C. accolens* (4) *C. pseudotuberculosis* (3) Untypable *corynebacterium* (2) *C. striatum* (1)
28	16	M	13 October 2022	14 October 2022	Fever, sore throat	HAdV-C (6)
29	39	M	17 October 2022	17 October 2022	Malaise, sore throat	*C. propinquum* (7) Untypable *corynebacterium* (3) *Moraxella catarrhalis* (17)
30	69	F	17 October 2022	18 October 2022	Fever, malaise, stuffy nose	EV-D68 (2889)
31	3	M	12 October 2022	19 October 2022	Fever, cough, stuffy nose	*Moraxella catarrhalis* (151)
32	41	M	18 October 2022	19 October 2022	Fever, malaise, sore throat, chills, joint pain	HAdV-C (2)
33	34	M	18 October 2022	21 October 2022	Fever, malaise, sore throat	*C. pseudotuberculosis* (2)
34	30	M	20 October 2022	21 October 2022	Fever malaise, sore throat, stuffy nose, cough, phlegm	HAdV-C (2) RSV-A (2)
35	42	F	20 October 2022	22 October 2022	Fever, malaise, sore throat, cough, nausea	*Moraxella catarrhalis* (3514)
36	42	M	21 October 2022	24 October 2022	Fever	Untypable *corynebacterium* (8) *C. accolens* (4) *C. striatum* (2) *C. propinquum* (1)
37	61	F	24 October 2022	24 October 2022	Sore throat	*C. aurimucosum* (2) *C. striatum* (2)
38	31	M	24 October 2022	25 October 2022	Fever, malaise, stomachache, nausea	HAdV-C (2) *C. accolens* (12) *C. minutissimum* (12) *C. pseudotuberculosis* (11) *C. striatum* (4) *C. aurimucosum* (2)
39	57	M	25 October 2022	26 October 2022	Malaise, sore throat, cough, diarrhea	HAdV-C (8)
40	66	F	27 October 2022	28 October 2022	Fever, diarrhea	*C. propinquum* (153) Untypable *corynebacterium* (31) *C. striatum* (6) *C. pseudotuberculosis* (4) *C. aurimucosum* (2)

HAdV-C, human adenovirus species C; RSV-A, respiratory syncytial virus subgroup A; HRV-A, human rhinovirus species A; EV-D; enterovirus species D. The numbers in the parenthesis are numbers of the short reads obtained from NGS analyses.

**Table 3:** Summary of pathogen profiles in the present cases.

Pathogens	Detection Rate (%)	Detection Cases
HAdV-C	40.0	(16/40)
RSV-A	15.0	(6/40)
HRV-A24	5.0	(2/40)
HRV-A61	2.5	(1/40)
HRV-A21	2.5	(1/40)
EV-D68	2.5	(1/40)
*C. accolens*	35.0	(14/40)
*C. pseudotuberculosis*	35.0	(14/40)
*C. striatum*	35.0	(14/40)
Untypable *corynebacterium*	27.5	(11/40)
*C. minutissimum*	25.0	(10/40)
*C. propinquum*	20.0	(8/40)
*C. aurimucosum*	15.0	(6/40)
*C. camporealensis*	7.5	(3/40)
*C. pseudodiphtheriticum*	7.5	(3/40)
*C. jeikeium*	5.0	(2/40)
*C. urealyticum*	2.5	(1/40)
*Moraxella catarrhalis*	12.5	(5/40)
*Staphylococcus aureus*	7.5	(3/40)
*Streptococcus pyogenes*	2.5	(1/40)

HAdV-C, human adenovirus species C; RSV-A, respiratory syncytial virus subgroup A; HRV-A, human rhinovirus species A; EV-D; enterovirus species D.

**Table 4:** Summary of non-pathogenic coryneforms in the present cases.

Species	Detection Rate (%)	Detection Cases
*C. segmentosum*	80.0	(20/25)
*C. kefirresidentii*	76.0	(19/25)
*C. tuberculostearicum*	72.0	(18/25)
*C. sanguinis*	52.0	(13/25)
*C. simulans*	52.0	(13/25)
*C. occultum*	40.0	(10/25)
*C. ciconiae*	28.0	(7/25)
*C. phocae*	28.0	(7/25)
*C. endometrii*	24.0	(6/25)
*C. kroppenstedtii*	20.0	(5/25)
*C. macginleyi*	16.0	(4/25)
*C. singular*	16.0	(4/25)

## Data Availability

Not applicable.

## Supplementary Materials

The following supporting information can be downloaded at: https://www.mdpi.com/article/10.3390/microorganisms11092142/s1, Table S1: Non-pathogenic coryneform detected in the present cases.

## References

[1] World Health Organization (2020). Novel Coronavirus–Japan (ex-China) 17 January 2020. Disease Outbreak News.

[2] NATIONAL INSTITUTE OF INFECTIOUS DISEASE. https://www.niid.go.jp/niid/ja/allarticles/infectious-diseases.html?start=84.

[3] Tago H., Kimura H., Kozawa K., Fujie K. (2006). Long-term observation of fogwater composition at two mountainous sites in Gunma Prefecture, Japan. Water Air Soil Pollut..

[4] Karako K., Song P., Chen Y., Tang W. (2020). Shifting workstyle to teleworking as a new normal in face of COVID-19: Analysis with the model introducing intercity movement and behavioral pattern. Ann. Transl. Med..

[5] Moriyama M., Hugentobler W.J., Iwasaki A. (2020). Seasonality of respiratory viral infections. Annu. Rev. Virol..

[6] Oluwole O.S.A. (2015). Seasonal influenza epidemics and El Ninos. Front. Public Health.

[7] Kuitunen I., Artama M., Mäkelä L., Backman K., Heiskanen-Kosma T., Renko M. (2020). Effect of social distancing due to the COVID-19 pandemic on the incidence of viral respiratory tract infections in children in Finland during early 2020. Pediatr. Infect. Dis. J..

[8] Zhou H., Møhlenberg M., Thakor J.C., Tuli H.S., Wang P., Assaraf Y.G., Dhama K., Jiang S. (2022). Sensitivity to vaccines, therapeutic antibodies, and viral entry inhibitors and advances to counter the SARS-CoV-2 Omicron variant. Clin. Microbiol. Rev..

[9] Ikuse T., Aizawa Y., Yamanaka T., Hasegawa S., Hayashi T., Kon M., Tamura T., Saitoh A. (2023). Comparison of Clinical Characteristics of Children Infected with Coronavirus Disease 2019 between Omicron Variant BA. 5 and BA. 1/BA. 2 in Japan. Pediatr. Infect. Dis. J..

[10] Piersiala K., Kakabas L., Bruckova A., Starkhammar M., Cardell L.O. (2022). Acute odynophagia: A new symptom of COVID-19 during the SARS-CoV-2 Omicron variant wave in Sweden. J. Intern. Med..

[11] Wölfel R., Corman V.M., Guggemos W., Seilmaier M., Zange S., Müller M.A., Niemeyer D., Jones T.C., Vollmar P., Rothe C. (2020). Virological assessment of hospitalized patients with COVID-2019. Nature.

[12] Wilson M. (2009). Bacteriology of Humans: An Ecological Perspective.

[13] Bosch A.A., Biesbroek G., Trzcinski K., Sanders E.A., Bogaert D. (2013). Viral and bacterial interactions in the upper respiratory tract. PLoS Pathog..

[14] Efstratiou A., Lamagni T. (2022). Epidemiology of Streptococcus pyogenes. Streptococcus pyogenes: Basic Biology to Clinical Manifestations [Internet].

[15] Tsoy D., Tirasawasdichai T., Kurpayanidi K.I. (2021). Role of social media in shaping public risk perception during COVID-19 pandemic: A theoretical review. Int. J. Manag. Sci. Bus. Adm..

[16] Vincent A.T., Derome N., Boyle B., Culley A.I., Charette S.J. (2017). Next-generation sequencing (NGS) in the microbiological world: How to make the most of your money. J. Microbiol. Methods.

[17] Takeuchi F., Sekizuka T., Yamashita A., Ogasawara Y., Mizuta K., Kuroda M. (2014). MePIC, metagenomic pathogen identification for clinical specimens. Jpn. J. Infect. Dis..

[18] Yamashita A., Sekizuka T., Kuroda M. (2016). VirusTAP: Viral genome-targeted assembly pipeline. Front. Microbiol..

[19] Kujiraoka M., Kuroda M., Asai K., Sekizuka T., Kato K., Watanabe M., Matsukiyo H., Saito T., Ishii T., Katada N. (2017). Comprehensive diagnosis of bacterial infection associated with acute cholecystitis using metagenomic approach. Front. Microbiol..

[20] Sayers E.W., Beck J., Bolton E.E., Bourexis D., Brister J.R., Canese K., Comeau D.C., Funk K., Kim S., Klimke W. (2021). Database resources of the national center for biotechnology information. Nucleic Acids Res..

[21] Oliveira A., Oliveira L.C., Aburjaile F., Benevides L., Tiwari S., Jamal S.B., Silva A., Figueiredo H.C., Ghosh P., Portela R.W. (2017). Insight of genus Corynebacterium: Ascertaining the role of pathogenic and non-pathogenic species. Front. Microbiol..

[22] Schlaberg R., Chiu C.Y., Miller S., Procop G.W., Weinstock G., Committee P.P., Professional Practice Committee and Committee on Laboratory Practices of the American Society for Microbiology, Microbiology Resource Committee of the College of American Pathologists (2017). Validation of metagenomic next-generation sequencing tests for universal pathogen detection. Arch. Pathol. Lab. Med..

[23] Chang S.Y., Lee C.N., Lin P.H., Huang H.H., Chang L.Y., Ko W., Chang S.F., Lee P.I., Huang L.M., Kao C.L. (2008). A community-derived outbreak of adenovirus type 3 in children in Taiwan between 2004 and 2005. J. Med. Virol..

[24] Ryan M.A., Gray G.C., Smith B., McKeehan J.A., Hawksworth A.W., Malasig M.D. (2002). Large epidemic of respiratory illness due to adenovirus types 7 and 3 in healthy young adults. Clin. Infect. Dis..

[25] Graham B.S. (2017). Vaccine development for respiratory syncytial virus. Curr. Opin. Virol..

[26] O’Callaghan-Gordo C., Bassat Q., Diez-Padrisa N., Morais L., Machevo S., Nhampossa T., Quintó L., Alonso P.L., Roca A. (2013). Lower respiratory tract infections associated with rhinovirus during infancy and increased risk of wheezing during childhood. A cohort study. PLoS ONE.

[27] Filho E.P., da Costa Faria N.R., Fialho A.M., de Assis R.S., Almeida M.M.S., Rocha M., Galvao M., Dos Santos F.B., Barreto M.L., Leite J.P.G. (2007). Adenoviruses associated with acute gastroenteritis in hospitalized and community children up to 5 years old in Rio de Janeiro and Salvador, Brazil. J. Med. Microbiol..

[28] Berk A.J. (2013). Adenoviridae. Fields Virol..

[29] Wold W.S., Ison M.G. (2013). Adenovirus. Fields Virology.

[30] Walsh M.P., Seto J., Liu E.B., Dehghan S., Hudson N.R., Lukashev A.N., Ivanova O., Chodosh J., Dyer D.W., Jones M.S. (2011). Computational analysis of two species C human adenoviruses provides evidence of a novel virus. J. Clin. Microbiol..

[31] Cervantes-Ortiz S.L., Zamorano Cuervo N., Grandvaux N. (2016). Respiratory syncytial virus and cellular stress responses: Impact on replication and physiopathology. Viruses.

[32] Hall C.B. (2001). Respiratory syncytial virus and parainfluenza virus. N. Engl. J. Med..

[33] Hendry R.M., Godfrey E., Anderson L.J., Fernie B.F., McIntosh K. (1985). Quantification of respiratory syncytial virus polypeptides in nasal secretions by monoclonal antibodies. J. Gen. Virol..

[34] Tsutsumi H., Onuma M., Suga K., Honjo T., Chiba Y., Chiba S., Ogra P. (1988). Occurrence of respiratory syncytial virus subgroup A and B strains in Japan, 1980 to 1987. J. Clin. Microbiol..

[35] Hendry R.M., Talis A.L., Godfrey E., Anderson L.J., Fernie B.F., McIntosh K. (1986). Concurrent circulation of antigenically distinct strains of respiratory syncytial virus during community outbreaks. J. Infect. Dis..

[36] NATIONAL INSTITUTE OF INFECTIOUS DISEASE. https://www.niid.go.jp/niid/ja/allarticles/surveillance/510-iasr/graphs/2293-iasrgv4.html.

[37] McIntyre C.L., Knowles N.J., Simmonds P. (2013). Proposals for the classification of human rhinovirus species A, B and C into genotypically assigned types. J. Gen. Virol..

[38] Oberste M.S., Maher K., Schnurr D., Flemister M.R., Lovchik J.C., Peters H., Sessions W., Kirk C., Chatterjee N., Fuller S. (2004). Enterovirus 68 is associated with respiratory illness and shares biological features with both the enteroviruses and the rhinoviruses. J. Gen. Virol..

[39] Calvo C., Cuevas M.T., Pozo F., García-García M.L., Molinero M., Calderón A., Gonzalez-Esguevillas M., Pérez-Sautu U., Casas I. (2016). Respiratory infections by enterovirus D68 in outpatients and inpatients Spanish children. Pediatr. Infect. Dis. J..

[40] Munywoki P.K., Koech D.C., Agoti C.N., Bett A., Cane P.A., Medley G.F., Nokes D.J. (2015). Frequent asymptomatic respiratory syncytial virus infections during an epidemic in a rural Kenyan household cohort. J. Infect. Dis..

[41] Matoba Y., Abiko C., Ikeda T., Aoki Y., Suzuki Y., Yahagi K., Matsuzaki Y., Itagaki T., Katsushima F., Katsushima Y. (2015). Detection of the human coronavirus 229E, HKU1, NL63, and OC43 between 2010 and 2013 in Yamagata, Japan. Jpn. J. Infect. Dis..

[42] Esposito S., Bosis S., Niesters H.G., Tremolati E., Begliatti E., Rognoni A., Tagliabue C., Principi N., Osterhaus A.D. (2006). Impact of human coronavirus infections in otherwise healthy children who attended an emergency department. J. Med. Virol..

[43] Bonizzoli M., Arvia R., Di Valvasone S., Liotta F., Zakrzewska K., Azzi A., Peris A. (2016). Human herpesviruses respiratory infections in patients with acute respiratory distress (ARDS). Med. Microbiol. Immunol..

[44] Jackson M.A., Sommerauer J.F. (2002). Human herpesviruses 6 and 7. Pediatr. Infect. Dis. J..

[45] Nhan T.-X., Parienti J.-J., Badiou G., Leclercq R., Cattoir V. (2012). Microbiological investigation and clinical significance of Corynebacterium spp. in respiratory specimens. Diagn. Microbiol. Infect. Dis..

[46] Dias M., Shreevidya K., Rao S.D., Shet D. (2010). Corynebacterium macginleyia rare bacteria causing infection in an immunocompromised patient. J. Cancer Res. Ther..

[47] Dangel A., Berger A., Konrad R., Sing A. (2019). NGS-based phylogeny of diphtheria-related pathogenicity factors in different Corynebacterium spp. implies species-specific virulence transmission. BMC Microbiol..

[48] Silva W.M., Dorella F.A., Soares S.C., Souza G.H., Castro T.L., Seyffert N., Figueiredo H., Miyoshi A., Le Loir Y., Silva A. (2017). A shift in the virulence potential of Corynebacterium pseudotuberculosis biovar ovis after passage in a murine host demonstrated through comparative proteomics. BMC Microbiol..

[49] Rosato A.E., Lee B.S., Nash K.A. (2001). Inducible macrolide resistance in Corynebacterium jeikeium. Antimicrob. Agents Chemother..

[50] NATIONAL INSTITUTE OF INFECTIOUS DISEASE. https://www.niid.go.jp/niid/ja/rs-virus-m/rs-virus-idwrs/11487-rsv-20220916.html.

