# Supplementary material for: Pathogen Profiles in Outpatients with Non-COVID-19 during the 7th Prevalent Period of COVID-19 in Gunma, Japan
Source: Microorganisms. 2023 Aug 24;11(9):2142. doi: 10.3390/microorganisms11092142 (PMC10536078; doi:10.3390/microorganisms11092142)
Supplement: Supplementary file 1 [file microorganisms-11-02142-s001.zip › microorganisms-2555709-supplementary.pdf]

Table S1: Non-pathogenic Coryneform detected in the present cases.

Case No.	Age	Gender	Onset date	Sampling date	Symptoms	Estimated pathogens
2	20	F	2022/9/19	2022/9/20	fever, malaise, headache	*Corynebacterium segmentosum* (73)<br>*Corynebacterium kefirresidentii* (42)<br>*Corynebacterium tuberculostearicum* (12)<br>*Corynebacterium sanguinis* (4)<br>*Corynebacterium endometrii* (2)
3	75	F	2022/9/17	2022/9/20	fever, malaise, cough, swollen throat	*Corynebacterium propinquum* (1176)<br>*Corynebacterium spp.* (200)<br>*Corynebacterium accolens* (120)<br>*Corynebacterium pseudotuberculosis* (69)<br>*Corynebacterium striatum* (52)
5	25	F	2022/9/19	2022/9/21	sore throat, headache	*Corynebacterium segmentosum* (72)<br>*Corynebacterium accolens* (6)<br>*Corynebacterium kefirresidentii* (4)<br>*Corynebacterium kroppenstedtii* (2)<br>*Corynebacterium tuberculostearicum* (2)<br>*Corynebacterium sanguinis* (2)<br>*Corynebacterium rouxii* (2)<br>*Corynebacterium ciconiae* (1)
6	31	M	2022/9/22	2022/9/24	fever, malaise, sore throat, headache	*Corynebacterium segmentosum* (150)<br>*Corynebacterium spp.*(31)<br>*Corynebacterium kefirresidentii* (12)<br>*Corynebacterium sanguinis* (6)<br>*Corynebacterium tuberculostearicum* (5)<br>*Corynebacterium simulans* (5)<br>*Corynebacterium ciconiae* (2)<br>*Corynebacterium canis* (2)<br>*Corynebacterium lactis RW2-5* (2)<br>*Corynebacterium glutamicum* (1)<br>*Corynebacterium occultum* (1)<br>*Corynebacterium stationis* (1)
7	56	F	2022/9/22	2022/9/24	fever, malaise, lymphoadenonopathy under the ear	*Corynebacterium segmentosum* (4)<br>*Corynebacterium tuberculostearicum* (2)<br>*Corynebacterium kefirresidentii* (2)
11	47	F	2022/9/26	2022/9/27	fever, malaise, headache, stuffy nose	*Corynebacterium segmentosum* (5)<br>*Corynebacterium kefirresidentii* (4)<br>*Corynebacterium rouxii* (2)

12	52	M	2022/9/28	2022/9/28	fever, cough, diarrhea	*Corynebacterium segmentosum* (7)
						Corynebacterium tuberculostearicum (5)
						Corynebacterium kefirresidentii (2)
13	46	F	2022/9/26	2022/9/28	fever, diarrhea	*Corynebacterium kefirresidentii* (4)
						Corynebacterium tuberculostearicum (2)
						Corynebacterium simulans (2)
						Corynebacterium segmentosum (1)
14	27	F	2022/9/24	2022/9/28	fever	*Corynebacterium macginleyi* (11)
						Corynebacterium sanguinis (10)
						Corynebacterium occultum (9)
						Corynebacterium kefirresidentii (4)
						Corynebacterium tuberculostearicum (2)
						Corynebacterium endometrii (2)
16	33	F	2022/9/30	2022/9/30	fever, malaise	*Corynebacterium kefirresidentii* (10)
						Corynebacterium segmentosum (9)
						Corynebacterium simulans (6)
						Corynebacterium tuberculostearicum (3)
17	46	M	2022/9/29	2022/9/30	sore throat	*Corynebacterium spp.* (27)
						Corynebacterium kefirresidentii (14)
						Corynebacterium segmentosum (12)
						Corynebacterium simulans (8)
						Corynebacterium tuberculostearicum (7)
						Corynebacterium lipophiloflavum (2)
						Corynebacterium occultum (1)
						Corynebacterium sanguinis (1)
						Corynebacterium glaucum (1)
18	15	M	2022/10/2	2022/10/3	fever, sore throat	*Corynebacterium segmentosum* (113)
						Corynebacterium kefirresidentii (46)
						Corynebacterium tuberculostearicum (30)
						Corynebacterium simulans (11)
						Corynebacterium occultum (11)
						Corynebacterium sanguinis (7)
						Corynebacterium spp. (4)
						Corynebacterium phocae (4)
						Corynebacterium ciconiae (3)
						Corynebacterium comes (2)
						Corynebacterium kroppenstedtii (1)
						Corynebacterium falsenii (1)

20	53	F	2022/10/3	2022/10/3	fever	*Corynebacterium segmentosum* (128)
						Corynebacterium sanguinis (7)
						Corynebacterium spp.(6)
						Corynebacterium tuberculostearicum (6)
						Corynebacterium endometrii (6)
						Corynebacterium singular (2)
						Corynebacterium occultum (2)
						Corynebacterium simulans (1)
						Corynebacterium phocae (1)
						Corynebacterium macginleyi (1)
						Corynebacterium flavescens (1)
23	63	M	2022/10/10	2022/10/11	sore throat	*Corynebacterium segmentosum* (140)
24	31	M	2022/10/10	2022/10/11	fever, malaise, sore throat, headache	*Corynebacterium spp.*(16)
						Corynebacterium tuberculostearicum (12)
						Corynebacterium kefirresidentii (8)
						Corynebacterium sanguinis (5)
						Corynebacterium phocae (3)
						Corynebacterium occultum (3)
						Corynebacterium ciconiae (2)
						Corynebacterium uterequi (2)
						Corynebacterium macginleyi (2)
						Corynebacterium endometrii (2)
						Corynebacterium simulans (1)
						Corynebacterium urogenitale (1)
25	54	M	2022/10/7	2022/10/11	fever, malaise, sore throat	*Corynebacterium kefirresidentii* (79)
26	34	F	2022/10/12	2022/10/12	fever, cough, sore throat	*Corynebacterium tuberculostearicum* (62)
						Corynebacterium simulans (19)
						Corynebacterium segmentosum (15)
						Corynebacterium singular (7)
						Corynebacterium occultum (6)
						Corynebacterium kroppenstedtii (2)
						Corynebacterium sanguinis (2)
						Corynebacterium sp. FDAARGOS 1242 (2)
						Corynebacterium endometrii (2)
						Corynebacterium phocae (1)
						Corynebacterium coyleae (1)
						Corynebacterium nuruki (1)
						Corynebacterium flavescens (1)
						Corynebacterium sp. 4H37-19 (1)

27	20	M	2022/10/13	2022/10/14	fever, malaise, sore throat, swollen throat	*Corynebacterium segmentosum* (27)<br>*Corynebacterium kefirresidentii* (12)<br>*Corynebacterium tuberculostearicum* (3)<br>*Corynebacterium ciconiae* (2)<br>*Corynebacterium imitans* (2)<br>*Corynebacterium spp.* (2)<br>*Corynebacterium simulans* (1)
28	16	M	2022/10/13	2022/10/14	fever, sore throat	*Corynebacterium kefirresidentii* (14)<br>*Corynebacterium kroppenstedtii* (10)<br>*Corynebacterium tuberculostearicum* (3)<br>*Corynebacterium occultum* (2)<br>*Corynebacterium sanguinis* (2)<br>*Corynebacterium segmentosum* (2)<br>*Corynebacterium simulans* (2)
29	39	M	2022/10/17	2022/10/17	malaise, sore throat	*Corynebacterium segmentosum* (8)<br>*Corynebacterium spp.* (3)<br>*Corynebacterium phocae* (1)
33	34	M	2022/10/18	2022/10/21	fever, malaise, sore throat	*Corynebacterium kefirresidentii* (12)<br>*Corynebacterium simulans* (10)<br>*Corynebacterium sanguinis* (4)<br>*Corynebacterium tuberculostearicum* (3)<br>*Corynebacterium kroppenstedtii* (2)<br>*Corynebacterium variabile* (1)<br>*Corynebacterium singular* (1)
36	42	M	2022/10/21	2022/10/24	fever	*Corynebacterium segmentosum* (10)<br>*Corynebacterium spp.* (8)<br>*Corynebacterium ciconiae* (3)<br>*Corynebacterium kalinowskii* (1)
37	61	F	2022/10/24	2022/10/24	sore throat	*Corynebacterium segmentosum* (6)<br>*Corynebacterium kefirresidentii* (4)
38	31	M	2022/10/24	2022/10/25	fever, malaise, stomach ache, nausea	*Corynebacterium segmentosum* (99)<br>*Corynebacterium kefirresidentii* (23)<br>*Corynebacterium tuberculostearicum* (21)<br>*Corynebacterium sanguinis* (11)<br>*Corynebacterium occultum* (9)<br>*Corynebacterium lipophiloflavum* (4)<br>*Corynebacterium singular* (3)<br>*Corynebacterium phocae* (3)<br>*Corynebacterium simulans* (2)<br>*Corynebacterium glutamicum* (2)<br>*Corynebacterium ciconiae* (1)<br>*Corynebacterium macginleyi* (1)

						Corynebacterium urogenitale (1)
						Corynebacterium endometrii (1)
40	66	F	2022/10/27	2022/10/28	fever, diarrhea	*Corynebacterium sp.* (31)
						Corynebacterium segmentosum (8)
						Corynebacterium occultum (5)
						Corynebacterium kefirresidentii (4)
						Corynebacterium tuberculostearicum (3)
						Corynebacterium sanguinis (3)
						Corynebacterium simulans (2)
						Corynebacterium phocae (2)
